# The crystalline style of molluscs: the nonlinear dynamics of a macroscale rotating structure in biology

**DOI:** 10.1098/rsif.2025.1232

**Published:** 2026-10-21

**Authors:** Sergio Testón-Martínez, Louise Lavictoire, Antonio G. Checa, C. Ignacio Sainz-Díaz, Julyan H. E. Cartwright

**Affiliations:** ^1^ Instituto Andaluz de Ciencias de la Tierra, IACT-CSIC, 18100 Armilla, Granada, Spain; ^2^ University of Granada, Granada, Spain; ^3^ Freshwater Biological Association, The Ferry Landing, Far Sawrey, Ambleside, Cumbria, LA22 0LP, UK; ^4^ Departamento de Estratigrafía y Paleontología, Universidad de Granada, 18071 Granada, Spain; ^5^ Instituto Universitario Carlos I de Física Teórica y Computacional, Universidad de Granada, 18071 Granada, Spain

**Keywords:** style, mollusc, fluid dynamics, mixing

## Abstract

We investigate the biophysics of crystalline style rotation in molluscs. We show that its efficient functioning as a type of food processor and mixer suggests a mechanism determined by the nonlinear physics of the variation in the rotation rate of the style. The crystalline style of molluscs is perhaps the only example above the cellular level of continuous rotation of a body part in animals.

## 1. Introduction

The only example that we are aware of in which there is free and complete rotation in a body part in the world of animals, or eukaryotes more generally, is the so-called crystalline style of molluscs (figure 1). Here, we investigate from the point of view of physics how variable style rotation speed may be key to how the style functions as a ‘food mixer’ of the bivalve digestive system. We analyse laboratory experiments on live juvenile freshwater mussels in order to extract data for time-series analysis of style rotation rates. We combine this work with mathematical modelling of a minimal model of style rotation to demonstrate the fluid-mechanical implications for its efficient biological functioning as a type of food processor and mixer.

**Figure 1.**
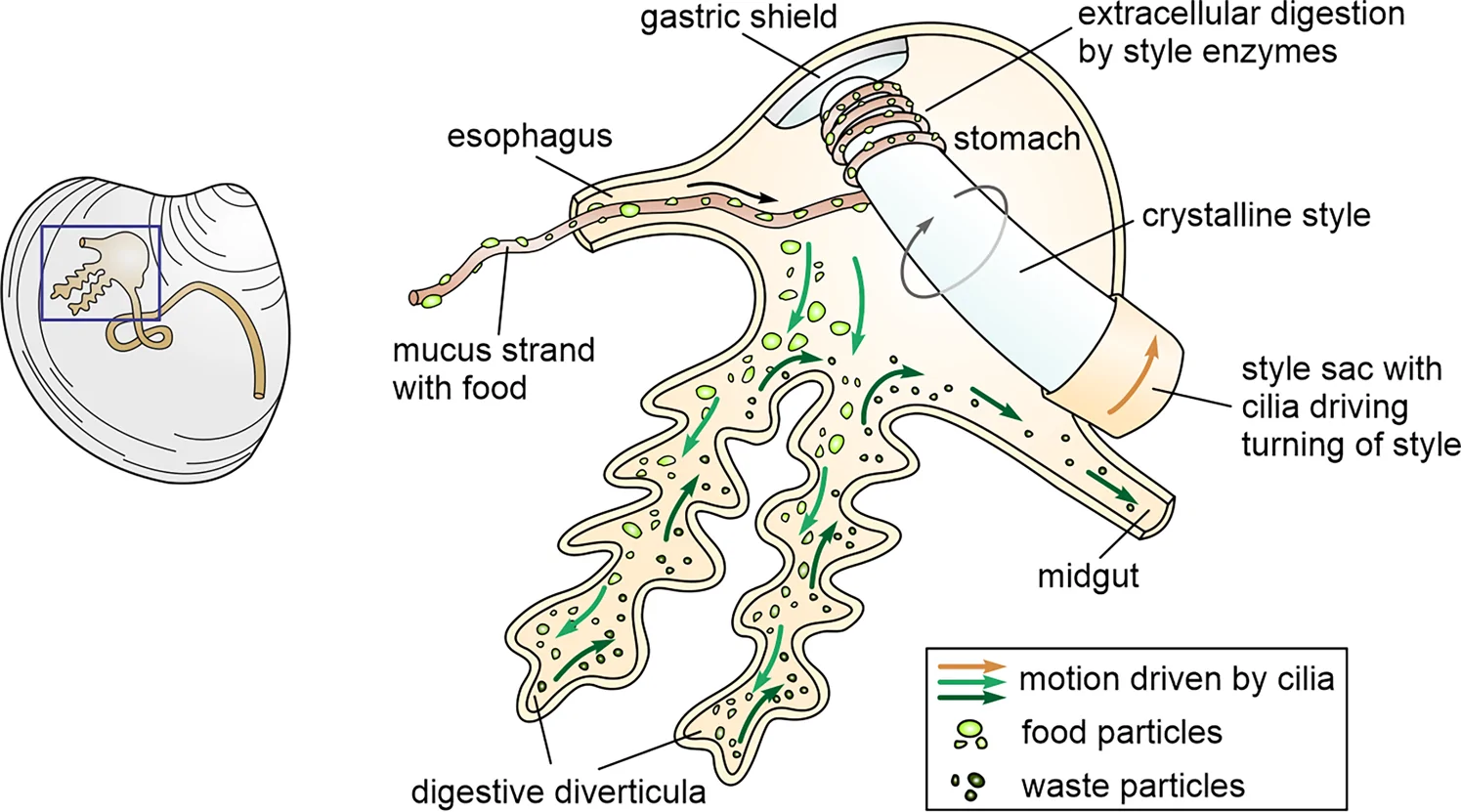
Diagrammatic sketch of the functioning of the crystalline style in the digestive system of a bivalve mollusc, drawn based on [1].

The crystalline style is an acellular hyaline gelatinous rod hosted in an invagination of the stomach called the style sac. The crystalline style is a central structure in the digestion of the animal and is found in the digestive system of certain gastropods and all bivalves except protobranchs. These have a so-called protostyle, which, contrary to the style, is unlaminated [2].

The style was discovered in 1686 by de Heide [3] who called it a *stylus cristallinus*, a name that has endured since then [4]. Other zoologists noted the presence of the style, but it was not thoroughly investigated until Poli (1791–1795) [5], who was also the discoverer of the gastric shield (see below). Coupin [6] and Mitra [7] arrived independently at the conclusion that the style was concerned with digestion, something already suggested by de Heide.

Styles are found either in a style sac completely or incompletely separated from the intestine, or the style may lie within the intestine itself [8]. The style projects from its sac into the stomach where it presses against a chitinous area of the stomach wall, referred to as the gastric shield [9,10]. The enzymes accumulated by the style are released upon rotation and abrasion of the style against the surface of the shield [11,12].

Rotation of the style causes mucous-bound material to be drawn from the oesophagus by the tip of the style, where the material is stirred and mixed with the enzymes released from the style [13]. In this way, the crystalline style is thought to help in digestion by reducing the size of food particles and directing them to the appropriate compartment for digestion [8,14–17]. Among other enzymes, the crystalline style releases amylase (starch digesting enzyme), cellulase and laminarinase [17,18]; a lipolytic enzyme was found by George [19]. In addition, the crystalline style provides an optimal environment for digestion by buffering ambient pH. Its physiological characteristics, including pH and dissolution, have been described by Hameed [20] and Judd [16].

All in all, stomach and style are hypothesized to act together as an organ of digestion and trituration [12].

## 2. Previous observations of the rotation of the style

There is not a great deal of published work on style rotation; even so, there is an amount of disagreement within the literature on how and when the style rotates. The first to consider the question was Nelson, in 1918 [14]. He writes

After nearly two years of experimenting, in which many individuals, both marine and fresh-water, were opened, I was able finally to cut through the stomach wall and expose the head of the style in such a way that the animal did not contract unduly, with consequent displacement of the alimentary canal.

As a result the style is not put under tension, but is free to rotate in the style sac, in spite of the fact that it forms a U-shaped bend in following the course of the intestine. To my surprise, I found this rotation to be quite rapid. In *Anodonta* kept at a constant temperature of 11.5${}^{\circ }$C prior to, and during the experiment, the maximum number of revolutions per minute was eleven. In *Modiolus*, at 25${}^{\circ }$C, the maximum number per minute was thirteen.

In all cases where I have had the revolving style under observation the motion is not continuous, but is interrupted by periods of inactivity, preceded by a gradual slowing down of the style. During these periods of cessation the cilia of the style sac themselves become greatly reduced in activity, After a period of rest the cilia again begin to beat actively, followed by a resumption of motion of the style. What the stimuli may be that cause the cessation and resumption of motion on the part of the style I have been unable thus far to determine. The presence or absence of food in the stomach itself seems to be of no consequence so far as this activity is concerned. It is possible that the large bristle-like cilia of the style sac may be under the control of the nervous system and respond to certain internal reflex stimuli called forth by the stage of digestion of the animal.

A more recent paper by Alyakrinskaya [17] compiled published data up to 2001. She writes

The main feature of crystalline styles is their capacity for rotation. Rotation takes place owing to the ciliary epithelium lining the style sac. The style rotates all along clockwise if viewed from the anterior end. The rate of rotation varies: 11 rotations/min in *Anodonta*, 13 in *Modiolus*, 20–40 in *Loripes*, and up to 90 in *Ostrea edulis* larvae.

It was reported that, in *Modiolus*, during digestion, the style rotated with intervals, rather than continuously.

It is believed that rotation takes place only during the style formation. However, the present author has never observed its movement in oysters, although hundreds molluscs were cut open. No style rotation was recorded in other molluscs [8].

The style rotation was always assigned a great significance when discussing its functions. The food material is pulled on the style head owing to rotation, and the food is more rapidly drawn in the stomach. In addition, rotation promotes mixing, grinding, and sorting the digestive tract contents. The dissolving style material containing enzymes is mixed with food and promotes its digestion.

Our aim here is to contribute towards clarifying some of these disagreements by both experimental observations of a model species, computer simulations and physical theory of the fluid dynamics of the crystalline style.

## 3. Analysis of experimental time series

Juvenile freshwater mussels (figure 2) are a good model organism in which to study the dynamics of the style because they are transparent for several months. We took videos with fluorescent microbeads and juvenile *Lampsilis siliquoidea* mussels to measure clearance rates and to visualize how the style works. As the juveniles consume the beads, they should light up as they pass through the digestive tract.

**Figure 2.**
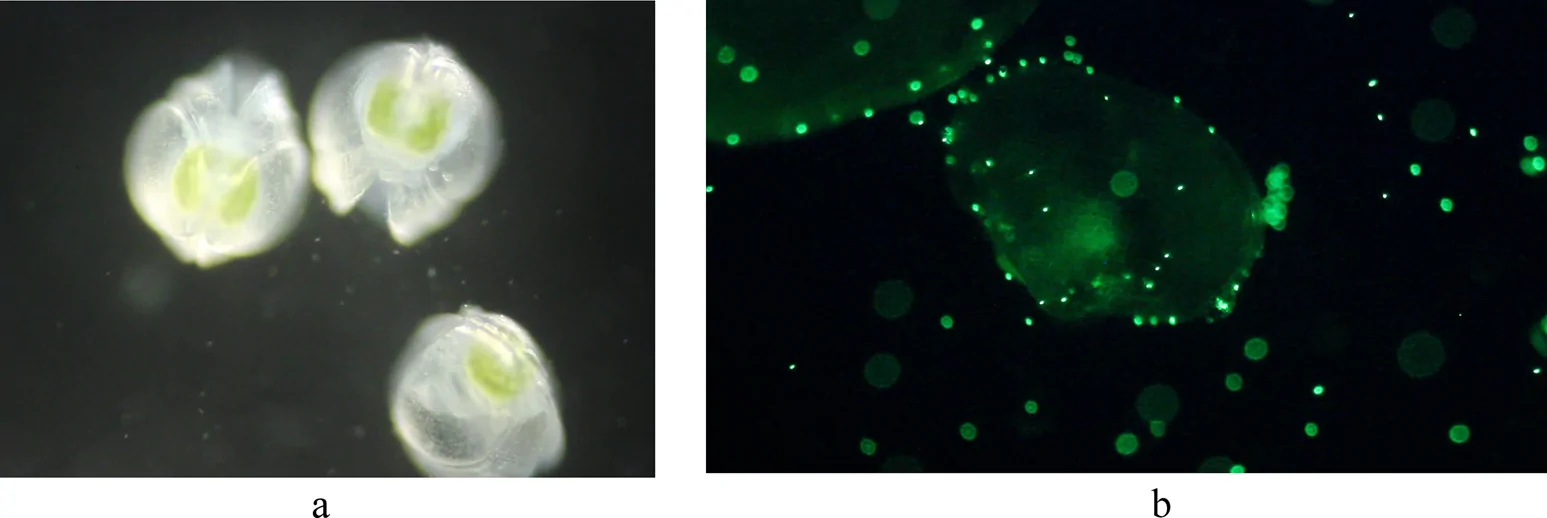
Juvenile freshwater mussels, *Lampsilis siliquoidea*. (a) The organism, *L. siliquoidea,* is approximately 180–200 µm newly excysted. The individuals used for the fluorescence videos were only a few days old and approximately 250 µm in size. (b) Still from microbead videos (supplementary material) used to analyse the style rotation.

In the images, there can be seen food particles in the intestine and gut together with the rotating style. The intestine passes through the heart and so the beating of the heart may provide pressure to the intestine, forcing food backwards and forwards with each contraction and relaxation.

Standard methods for glochidial encystment using an innoculation bath with their host fish, largemouth bass (*Micropterus salmoides*), were used, as described in Patterson *et al.* [21]. Juvenile *L. siliquoidea* were collected after excystment and placed into a bucket system as described in Barnhart [22]. Juveniles were then removed for videography, as required, and returned to their system afterwards.

We extracted time series of the velocity of the style from the experimental videos (figure 3). Eight videos at 1920 × 1080 resolution and 30 fps were analysed, each centred on a different individual for the study of style motion; one of these videos (13 s in duration, included as supplementary material as ‘Fluorescent mussel style rotating.mp4’) was used to extract the frequency time series listed in table 1 and employed in the simulations of §4. To perform this analysis, we located the coordinates of the style whose rotation we wanted to analyse, taking the bottom-left corner of the videos as the origin. The rotation of the crystalline style cannot be measured directly from the videos because of the limited spatial resolution of the fluorescence images. Instead, we use the periodic variation in fluorescence intensity as a proxy for rotational motion (figure 3*a*). The style rotates continuously while showing repeatable intensity maxima, which we interpret as successive rotational cycles. The fluorescence signal was extracted from a fixed rectangular region of interest (ROI) of 160 × 140 pixels centred on the crystalline style. The ROI was kept fixed throughout each video, and no explicit stabilization was applied, although motion was limited during the analysed sequences. The intensity signal was computed as the average pixel value within this region for each frame. Since the analysis focuses on peak timing rather than amplitude, no normalization of the signal was applied. We calculated the average illumination of the pixels in the area whose coordinates we had provided, obtaining a series of intensities, one value for each frame. Peaks in this intensity signal were identified manually using an interactive selection procedure. This was preferred because the signal is non-sinusoidal and often exhibits secondary local maxima within a single cycle, which can lead to over-detection with simple automated methods. Manual selection ensures one dominant peak per rotation. The times at which a peak maximum in illumination occurs are thus the times when a complete rotation took place (figure 3*b*). Inspection of the corresponding video frames at successive maxima shows similar visual configurations of the style, consistent with one full rotation between peaks.

**Figure 3.**
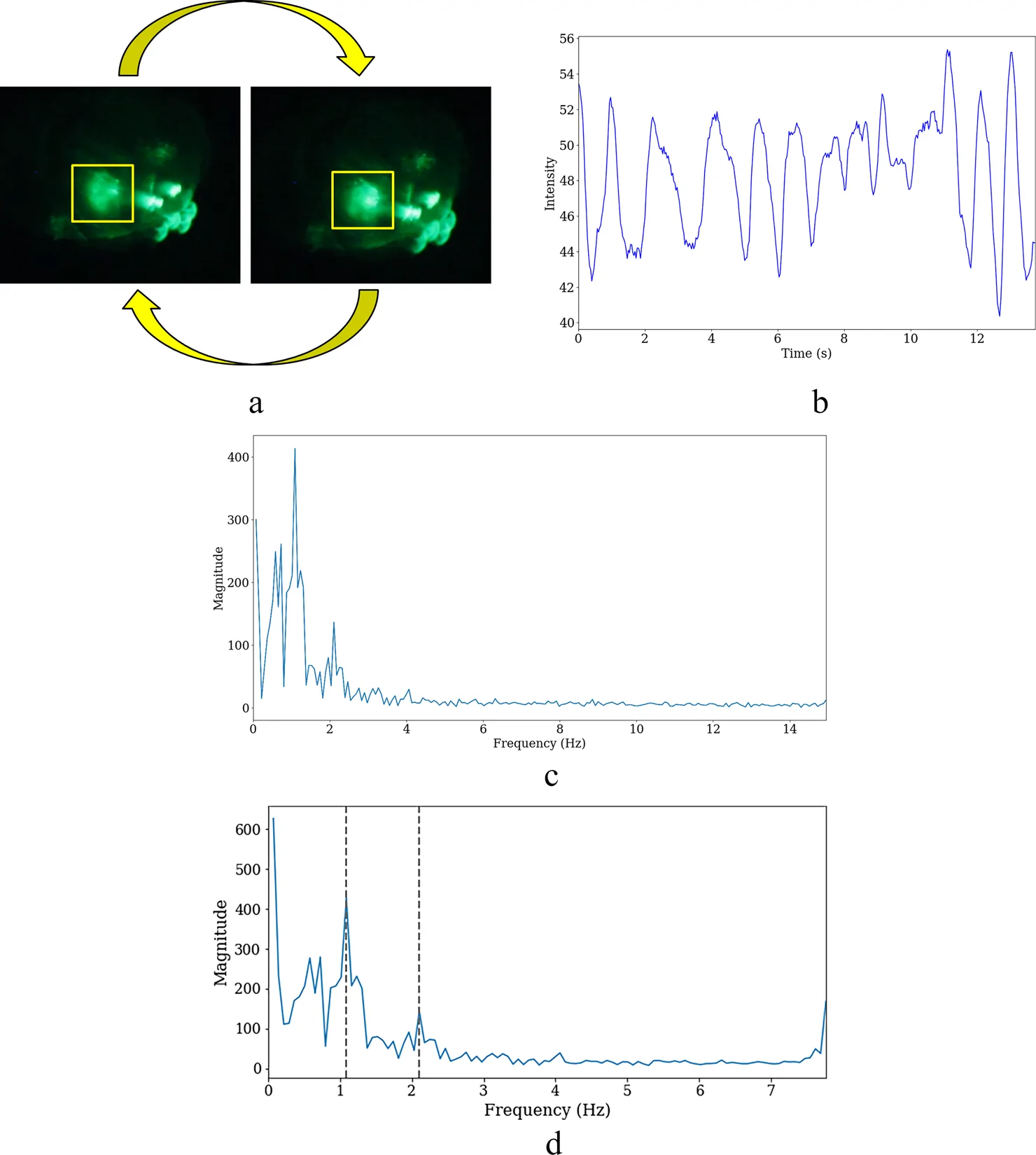
Time-series analysis of the microbead experimental videos. (a) Two frames showing the change in the illumination of the style through each rotation. (b) Variation of image intensity over time used to obtain the rotation rate. (c) Fourier transform of one video. (d) Fourier transform of the sum of five videos. Peaks are at 1.08 and 2.10 Hz. Sampling rate $f_{s}=29.97\;\mathrm{Hz}$ and frequency resolution Δf=0.073Hz.

**Table 1.** An example of the number and magnitude of the intensity of the style each rotational maximum, as well as the time it occurs and the period of rotation of spin.

peak	intensity	time (s)	period (s)
1	53.48	0.00	0.00
2	52.42	0.94	0.94
3	51.31	2.21	1.27
4	51.88	4.16	1.95
5	51.48	5.43	1.27
6	51.01	6.53	1.11
7	50.36	7.75	1.22
8	51.34	8.37	0.62
9	52.88	9.13	0.76
10	51.85	10.68	1.54
11	55.37	11.12	0.44
12	52.63	12.09	0.98
13	55.22	13.04	0.94

In figure 3, we can observe the periodic form of the rotation of the style and the intensity maxima mentioned. In addition to understanding the type of movement that the style follows, we can go a step further and look at the time between each intensity maximum. This is seen to vary; in other words, the rotations have different durations. In figure 3*b*, we can notice that the style rotates more slowly at the beginning of the video, it accelerates towards the middle and slows down again in the last seconds. Fourier transforms allow signals to be transformed between the time and the frequency domain. Thus, to test this variability, Fourier transforms were applied to the entire array of intensities. We see that the Fourier transform (figure 3*c*) does not show a single frequency peak; rather, there is a broad band of frequencies with several discernible maxima. This implies that the rotational speed of the style is not constant, and the rotational frequency varies. As these analyses refer to a single video, they have been repeated for the rest of the observations. Summing five Fourier frequency spectra found for each of the videos, we obtain the plot shown in figure 3*d*. From the summed data in figure 3*d*, we see that is a broad band of frequencies as the style rotates at a changing rate in every observation; the two largest peaks are at 1.08 and 2.10 Hz.

We can observe this variability in table 1, which lists both the maximum intensity and the period of rotation values, together with the spectrogram in figure 4*A*, and the histogram in figure 4*B* that represents the evolution of the period through time. To quantify this variability further, we analysed the distribution of inter-peak periods (excluding the first interval). The mean period is 1.09 s with a standard deviation of 0.39 s, giving a coefficient of variation of 0.36, indicating non-uniform rotation. To assess robustness, we compared the manually selected peaks with those obtained using an automated peak detection algorithm (scipy.signal.find_peaks) with a minimum peak separation of 0.5 s and a prominence threshold of 0.3. The resulting period statistics were similar between methods: mean period 1.10 s (manual: 1.09 s), standard deviation 0.45 s (manual: 0.39 s) and coefficient of variation 0.41 (manual: 0.36). This indicates that the inferred rotation dynamics are not sensitive to the peak detection method.

**Figure 4.**
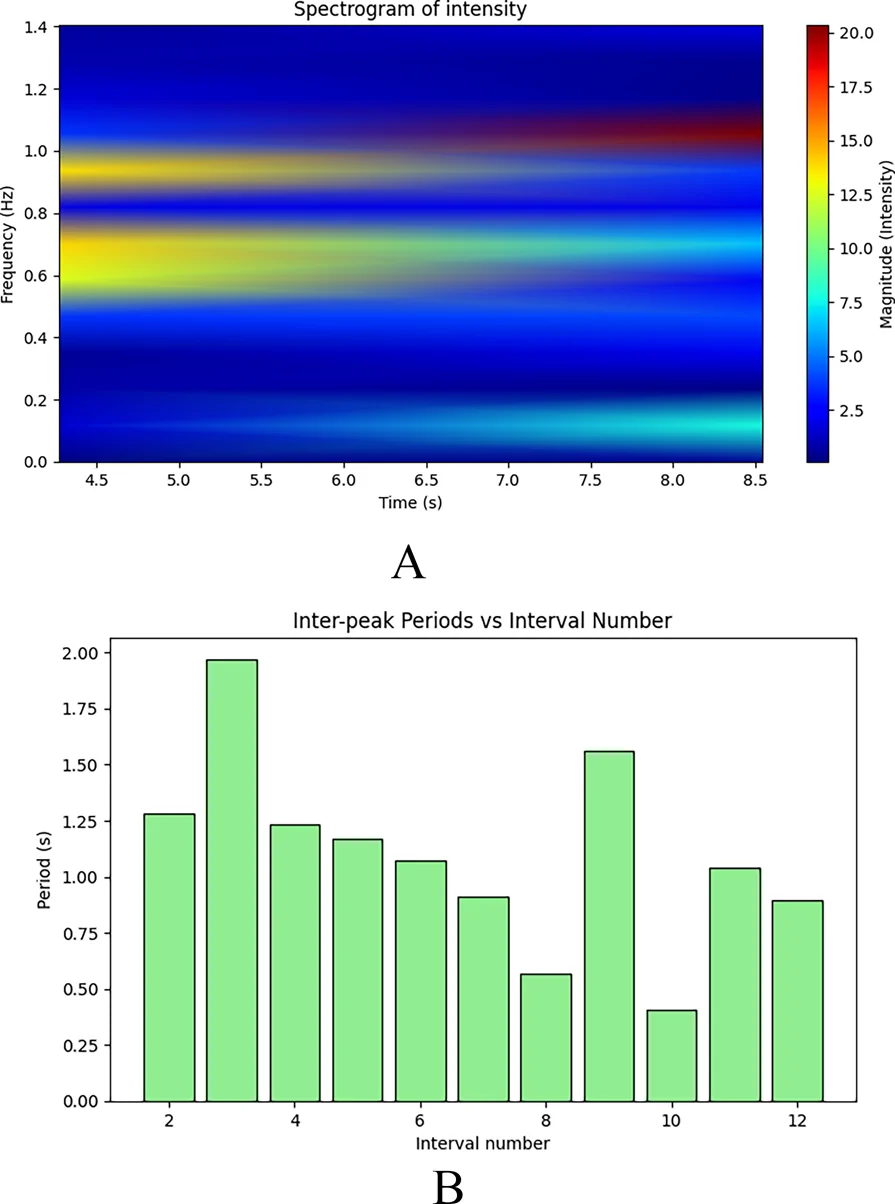
(A) Spectrogram of the intensity showing the variation of the frequency of rotation of the style over time. (B) Representation of the different periods for each rotation of the style during 13 s.

## 4. Biological fluid mechanics of the style

We have shown that the rotation velocity varies in the observational data. In order to understand why this variable rotation rate should be important for the organism, we need to call upon the fluid dynamics of slow flows.

The measure of how fast or slow a flow is, is indicated by its Reynolds number. Unlike for turbulent flows at high Reynolds numbers, mixing in laminar flows at low Reynolds number operates quite differently. At high Reynolds numbers, a fluid has inertia: when you stop stirring your tea, the tea keeps moving for some time on its own. At low Reynolds number, however, fluid friction wins out against inertia, and fluid motion ceases as soon as driving stops. Since one of the factors in the Reynolds number is a characteristic size, we can immediately understand that fluid inertia is more important for digestion in an elephant than in a mouse. A human stomach is on the cusp of where fluid inertia ceases to operate [23].

In the style, the relevant Reynolds number is

$$Re=ND^{2}/\nu ,$$

where N denotes style rotations per second, D denotes style diameter and ν denotes the kinematic viscosity of the fluid. From the data we have analysed, the juvenile *L. siliquoidea* style has the highest peak rotation rate at approximately once per second; N=1 Hz, and the range is approximately 0.5≲N≲2.5 Hz. From the earlier observations we have reported, this range is similar across species; there is anecdotal discussion of higher style rotation rates up to nearly 10 Hz. Further, the kinematic viscosity of water at 20ºC is $\nu =10^{-6}$ m${}^{2}$ s^–1^; the stomach adds enzymes and gastric mucus. There are not many measurements of mollusc mucus viscosity. Krings & Gorb [24] measured the viscosity of a terrestrial gastropod *Lissachatina fulica* saliva as 1.99 mPa s^–1^, twice that of water. Denny & Gosline [25] measured the pedal mucus of another terrestrial gastropod, *Ariolimax columbianus*, as 3000–5000 mPa s^–1^. Let us then take a wide range $2\times 10^{-6}<\nu <5\times 10^{-3}$ m^2^s^–1^ for the possible kinematic viscosity, between 2 and 5000 times that of water. The style diameter varies between species and increases with the age of the mollusc. The lower bound is determined by the size of a developing juvenile, and so can be arbitrarily small at the beginning of style development. The upper limit is given by the largest specimens of the largest species; Yonge [26] looked at *Tridacna derasa* and *Strombus gigas* and reported values up to 6 mm. We have not found values reported for *Tridacna gigas*, but that species, the largest extant bivalve mollusc, might be expected to have yet larger styles. From the well-attested values, we obtain a range of Reynolds numbers for the style across species and organism sizes between Re=0 and $O(10^{2})$ (table 2). For the lower end of these values, inertia does not play a role in the mixing inside the molluscan stomach. For the experiments we report here, a juvenile *L. siliquoidea* is on average 1 mm in size after 69 days [22]; the style within it will measure approximately 0.045–0.05 mm at this stage, so the maximum Reynolds number in such juvenile *L. siliquoidea* is approximately Re=0.5 to $2.5\times 10^{-3}$ in this inertialess range.

**Table 2.** Maximum Reynolds number values based on measured values of the style diameter and rotational velocity and cited values of mucal viscosity [24,25].

	diameter	rotation rate	kinematic viscosity	Reynolds number
style	D, mm	N, Hz	ν, m${}^{2}$ s^–1^$\times 10^{-6}$	$Re=ND^{2}/\nu$
across all species	0–6	0.0–10	2–5000	0–$O(10^{2})$
for these experiments	0.045–0.05	0.5–2.5	2–5000	(0.5–2.5)$\times 10^{-3}$

Two-dimensional fluid flows at low Reynolds numbers have the mathematical structure of a set of two-dimensional ordinary differential equations. The dynamical behaviour of these varies depending on whether there is time dependence, or not. In the case without time dependence, all trajectories are regular, which is what the name *laminar* flow implies. However, the time-dependent case is different; here, a new type of dynamics, so-called deterministic chaos, is seen, in which trajectories can exhibit great topological complexity. The importance of this chaos for fluid mixing was only noted in the 1980s, when the phenomenon was dubbed chaotic advection [27]. Chaotic advection is a phenomenon in fluid dynamics wherein passive tracers, such as particles or biological organisms, experience highly convoluted trajectories owing to complex flow patterns. Unlike simple laminar flows, which exhibit smooth and predictable streamlines, chaotic advection arises in systems with time-dependent velocity fields, leading to intricate mixing [28].

We can use simulations to study the mixing chaotic trajectories caused by this variation. We use the journal bearing flow as a minimal model of the crystalline style. The journal-bearing model consists of two eccentric cylinders representing the stomach (outer cylinder, radius $R_{1}$) and the crystalline style (inner cylinder, radius $R_{2}$), as well as a driving protocol N(t) governing the rotation frequency of the inner cylinder. These parameters determine the time-dependent forcing used in the simulations [29]. This corresponds to a schematic of a transversal section through the stomach of the molluscan digestive system (the outer cylinder) with the style within it (the inner cylinder). Some of the geometric characteristics—the eccentricity, size ratios and gap widths— must necessarily be estimated owing to the limited experimental data on the detailed geometry.

The journal bearing flow may then be viewed as a minimal model of a two-dimensional section through the crystalline style within the stomach of the organism—cf. figure 1—where the outer cylinder represents the stomach and the inner cylinder the style. A two-dimensional section is a reasonable approximation when the style is approximately cylindrical; discounting the complexities of the end effects, what goes on in the middle will be largely two-dimensional. The actual geometry of the stomach is more complicated than a cylinder, of course, but the offset represents the important aspect that the stomach–style system is not symmetrical under rotation. The journal bearing flow may be solved analytically. If the rotation velocity is maintained constant, then the system is a two-dimensional time-independent flow, which is regular, but if the rotation velocity varies, the system is a two-dimensional time-dependent flow, which has chaotic trajectories.

A repeated realization of the rotation is shown in figure 5. If the velocity is not constant, we can see that a single fluid particle has covered almost every point of the area available to it (figure 5*a*). The time-dependent driving protocol N(t) used in the simulations is constructed from the experimentally inferred rotation dynamics. Specifically, the instantaneous rotation rate is obtained from the sequence of inter-peak intervals in the intensity signal (figure 3*b*). This yields a discrete set of rotation periods, which are converted into a piecewise-constant angular velocity. To generate a sufficiently long time series for the simulations, this experimentally derived sequence is extended periodically by concatenating it with itself. In this way, the driving preserves both the amplitude variability and the characteristic time scales observed in the experiments. As a control, we also consider a constant rotation rate corresponding to the mean experimental period, ⟨T⟩=1.09s, i.e. N=1/⟨T⟩≈0.92Hz. In the case where the velocity is constant, a particle simply circles near the borders of the outer cylinder and does not mix completely like in the previous cases (figure 5*b*). Finally, we note that the initial position of the particle plays a crucial role in determining its ability to mix within the system. As illustrated in the case of figure 5*c*, a particle released too close to the limit zone becomes confined to a region of nearly closed streamlines and is therefore unable to escape into the main flow. Consequently, it remains isolated and does not mix with the rest of the domain.

**Figure 5.**
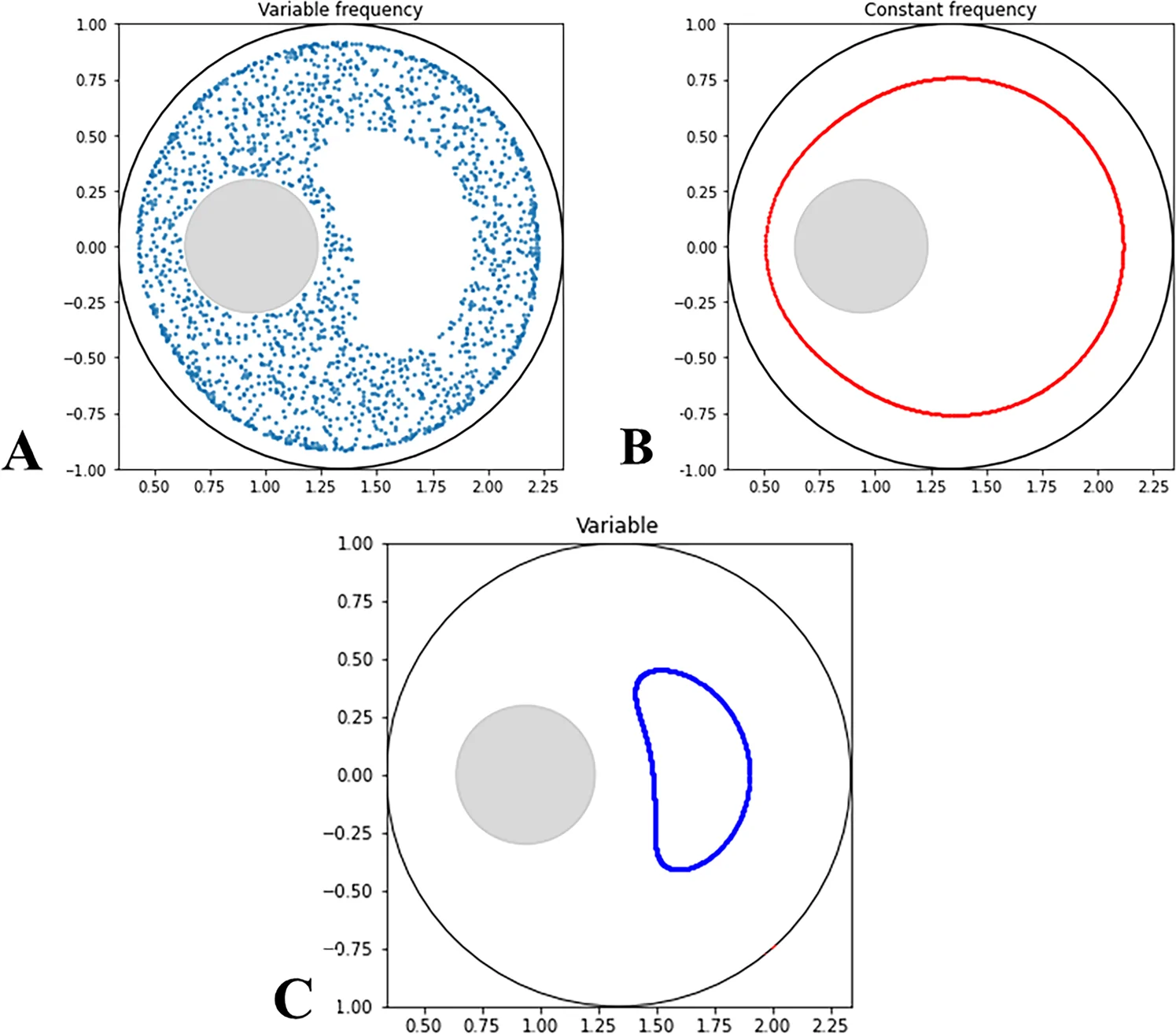
Journal bearing flow simulations. The flow is between two cylinders, marked as the inner filled circle and the outer open circle; $R_{1}/R_{2}=3.33$ for (a) variable angular velocity based on the frequencies obtained experimentally (initial particle position (0.75,0.5)). (b) A simulation with constant velocity. (c) Change in particle position (2.0,0.0).

Mixing efficiency may be quantified using both dynamical and spatial metrics. The finite-time Lyapunov exponent, computed via the Benettin algorithm with periodic renormalization of an infinitesimal perturbation, provides a measure of exponential stretching. As shown in figure 6, the simulation with variable frequency yields a significantly higher asymptotic exponent (λ≈0.43) compared to the constant case (λ≈0.01), indicating strong chaotic advection in the former and near-regular dynamics in the latter. Complementarily, spatial mixing is assessed through a coverage metric based on a coarse-grained occupancy of the domain using a two-dimensional histogram. The first case reaches a substantially larger explored fraction of phase space (coverage ≈0.421) than the constant case (coverage ≈0.077).

**Figure 6.**
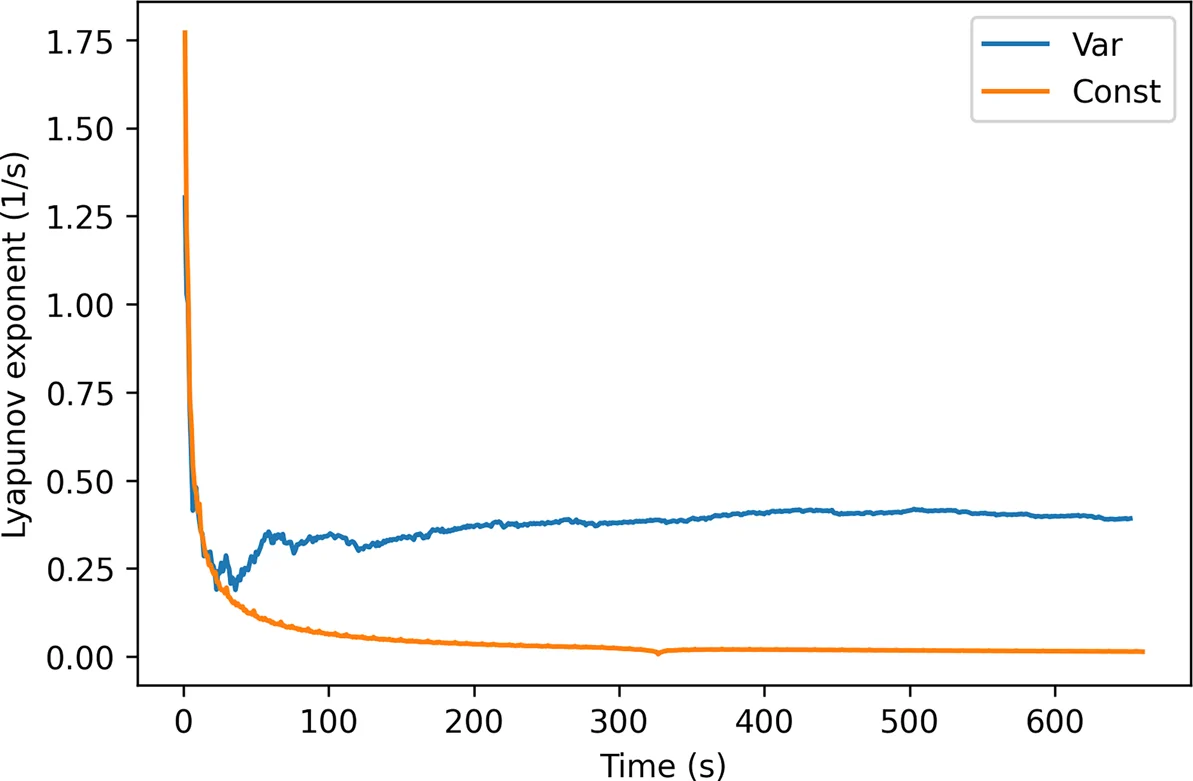
Time evolution of the finite-time Lyapunov exponent λ(t) for the variable (var) and constant (const) frequency simulations. The exponent is computed using the Benettin algorithm with continuous renormalization of the perturbation vector.

## 5. Discussion and conclusion

The implication of this physics for the crystalline style is that if the rotation speeds up and slows down, or stops and starts, as has been previously observed, the mixing will be greatly enhanced compared to if a constant rotation were maintained. This would clearly be of benefit to a digestive system that is operating under the physics of low-Reynolds-number flow. In style rotation, as indicated in figure 1, the driving is provided by motile cilia with a whip-like action, of the 9+2 type (see below). These are arranged around the axle of the structure to provide propulsion to the style via viscous hydrodynamic coupling. The style has evolved to use the coordinated linear, whip-like driving of cilia on a cellular level to propel continuous rotation, in what is to our knowledge a unique arrangement. As the style speeds up and slows down, we anticipate that these velocity changes of the style should arise from the ciliar motion similarly speeding up and slowing. It should be of interest to observe this process *in vivo*.

There is another instance of continuous rotation in the mollusc: the pearl. Some pearls are known to rotate within their pearl sac [30]. The pearl, however, is not a body part or an organ, but is an object biomineralized by a host organism in response to a source of irritation in its tissues. Pearl rotation, it has been proposed, may occur driven by the fluid mechanics of pearl growth itself [31], and pearl rotation, unlike style rotation, has no biological function.

In biology, the continuous rotation of part of an organism is ubiquitous at the cell scale, with rotating organelles: the flagellum of bacteria, which is a type of rotary engine [32], and ATP synthase, which has been called ‘a marvellous rotary engine of the cell’ [33]. Even at the cellular level, however, biology sometimes uses other means to the same effect; compare the prokaryotic flagellum with the eukaryotic cilium and flagellum, which achieves motion by a whip-like action (in so-called 9+2 cilia and flagella) [34] and in a few cases (motile 9+0 cilia) [35] the same type of motion as the bacterial flagellum, not by rotation, but rather by whirling, as a cowboy whirls a rope [36]. Above the cellular level, however, the type of biological movement generally found is that of partial rotation, as in our skeletal joints [37]. Reciprocating motion at a joint may be turned to continual forward propulsion, as in walking.

The reasons for the general absence of macroscale free rotation in biology certainly include the anatomical problem of how the axle of a biological wheel might be arranged [38]. The bacterial flagellum and ATP synthase show that it is possible to have a biological wheel on the microscale, but its scaling up seems to present very serious difficulties for biology. On the microscale, the torque is provided by motor proteins, but how might a larger-scale structure be driven? The crystalline style of the mollusc is a macroscopic example; the only macroscopic instance of continuous rotation of an organ or body part of which we are aware. The rotation of the style at a variable speed suggests a mechanism by which variable rotation may enhance mixing of the stomach contents so that food particles may be efficiently processed by the organism. It is instructive to compare this with stomach mixing in other organisms. In humans and many other mammals, stomach mixing is performed instead by peristaltic movements of the stomach walls [23]. In low-Reynolds-number environments, these must move in a cyclical fashion, and not merely in reciprocating motions that would merely lead to cycles of mixing and unmixing [39]. The mollusc may, perhaps uniquely in nature, have evolved a different strategy for its stomach, using continuous rotation of an impeller—the style—in a fashion similar to many man-made fluid mixers.

## Supplementary Material



## Data Availability

The datasets supporting this article are provided as supplementary material. These include fluorescence and non-fluorescence microscopy videos of the molluscs (supplementary material, files 1, 2 and 6–10) and Python scripts used for data processing, Fourier analysis and numerical simulations (supplementary material, files 3–5). All data and code necessary to reproduce the findings of this study are included within the supplementary material accompanying this article. Supplementary material is available online [40].

## Declaration of AI use

AI-assisted technologies were used in a limited manner to support the development of computational code. These tools were used to assist with programming syntax and minor coding tasks during the development of the simulations. All generated code was carefully reviewed, tested and validated by the authors. The scientific design, analysis, interpretation of results and conclusions are entirely the authors' own work. The authors take full responsibility for the accuracy and integrity of all content.
