# Supplementary material for: The crystalline style of molluscs: the nonlinear dynamics of a macroscale rotating structure in biology
Source: J R Soc Interface. 2026 Oct 21;23(243):20251232. doi: 10.1098/rsif.2025.1232 (PMC13682894; doi:10.1098/rsif.2025.1232)
Supplement: Supplementary file 1 [file rsif.2025.1232.zip › rsif.2025.1232/rsif20251232_si_011.pdf]

ESM File 1: Freshwater mussel style rotating.mp4

Title: Freshwater mussel style rotating

Caption: Video of a juvenile freshwater mussel. Lampsilis siliquoidea.

ESM File 2: Fluorescent mussel style rotating.mp4

Title: Fluorescent mussel style rotating

Caption: Example video of a freshwater mussel style rotating using fluorescent microbeads to visualize the rotation of the style.

ESM File 3: intensity_fourier.py

Title: Intensity and Fourier frequencies calculation code

Caption: Python code that extracts video intensity values and computes Fourier frequencies for a crystalline style.

ESM File 4: fourier_sum.py

Title: Fourier frequency summation code

Caption: Python code that is used to sum the Fourier frequencies from various videos obtained through the program above.

ESM File 5: journal_bearing.py

Title: Journal bearing simulation code

Caption: Python code used to simulate a journal bearing and calculate mixing metrics.

ESM File 6: Cohort227_Style1_cut1.mp4

Title: Processed fluorescent mollusc video #1

Caption: First microscopy video of the mollusc from which intensity values and frequencies were obtained.

ESM File 7: Cohort227_Style1_cut2.mp4

Title: Processed fluorescent mollusc video #2

Caption: Second microscopy video of the mollusc from which intensity values and frequencies were obtained.

ESM File 8: Cohort227_Style2_cut1.mp4

Title: Processed fluorescent mollusc video #3

Caption: Third microscopy video of the mollusc from which intensity values and frequencies were obtained.

ESM File 9: Cohort227_Style2_cut2.mp4

Title: Processed fluorescent mollusc video #4

Caption: Fourth microscopy video of the mollusc from which intensity values and frequencies were obtained.

ESM File 10: Cohort227_Style3_cut.mp4

Title: Processed fluorescent mollusc video #5

Caption: Fifth microscopy video of the mollusc from which intensity values and frequencies were obtained.
